# Epidemiological trends and novel prognostic evaluation approaches of patients with stage II-IV colorectal neuroendocrine neoplasms: A population-based study with external validation

**DOI:** 10.3389/fendo.2023.1061187

**Published:** 2023-02-01

**Authors:** Fuqiang Zhao, Liling Huang, Zhijie Wang, Fangze Wei, Tixian Xiao, Qian Liu

**Affiliations:** ^1^ Department of Colorectal Surgery, National Cancer Center/National Clinical Research Center for Cancer/Cancer Hospital, Chinese Academy of Medical Sciences and Peking Union Medical College, Beijing, China; ^2^ Department of Medical Oncology, National Cancer Center/National Clinical Research Center for Cancer/Cancer Hospital, Chinese Academy of Medical Sciences and Peking Union Medical College, Beijing Key Laboratory of Clinical Study on Anticancer Molecular Targeted Drugs, Beijing, China

**Keywords:** colorectal neuroendocrine neoplasms, incidence, survival, nomogram, SEER, competing risk analysis

## Abstract

**Objective:**

This study aimed to clarify the incidence trend of all-stage colorectal neuroendocrine neoplasms (CRNENs), overall survival (OS), and disease-specific survival (DSS) of patients with stage II-IV CRNENs, and to establish relevant nomograms for risk stratification.

**Methods:**

Among all patients diagnosed with CRNENs in the Surveillance, Epidemiology, and End Results (SEER) database from 1975 to 2019, temporal trends in incidence were assessed. Clinical data of 668 patients with stage II-IV CRNENs from 2010 to 2016 were extracted for survival analysis. Patients were randomly divided into a training cohort and a validation cohort at a ratio of 7:3. Univariate and multivariate cox regression analyses were utilized to identify independent prognostic factors affecting OS outcomes. Competing risk analysis was applied to investigate risk factors related to the DSS of CRNENs. Two nomograms specifically for OS and DSS were developed for patients with stage II-IV CRNENs, their prognostic capabilities were evaluated using calibration curves, receiver operating characteristic (ROC) curves, the time-dependent area under the curve (AUC), and decision-curve analysis (DCA). Our hospital’s independent cohort of 62 patients with CRNENs was used as the external validation cohort.

**Results:**

In the period of 1975-2019, the incidence of CRNENs increased steadily with an annual percentage change (APC) of 4.50 (95% confidence interval [CI]: 3.90–5.11, P < 0.05). In total, 668 patients with stage II-IV CRNENs were included in the survival analysis from 2010 and 2016. Independent adverse prognostic factors for both OS and DSS of CRNENs prior treatment included grade III/IV (HR for OS: 4.66, 95%CI: 2.92-7.42; HR for DSS: 4.79, 95%CI: 4.27-5.31), higher TNM stage ([stage III vs stage II] HR for OS: 2.22, 95%CI: 1.25-3.94; HR for DSS: 2.69, 95%CI: 1.96-3.42. [stage IV vs stage II] HR for OS: 3.99, 95%CI: 2.03-7.83; HR for DSS: 4.96, 95%CI: 4.14-5.78), liver metastasis (HR for OS: 1.61, 95%CI: 1.03-2.51; HR for DSS: 1.86, 95%CI: 1.39-2.32), and brain metastasis (HR for OS: 4.57, 95%CI: 1.66-12.58; HR for DSS: 5.01, 95%CI: 4.15-5.87). Advanced age was also identified as a risk factor for OS (HR: 2.03, 95%CI: 1.5-2.76) but not DSS. In terms of treatment, surgery can significantly prolong OS (HR: 0.62, 95%CI: 0.44-0.86) and DSS (HR: 0.67, 95%CI: 0.29-1.05), but chemotherapy and radiation failed to show significance. The respective nomograms for OS and DSS for stage II-IV CRNENs demonstrated high accuracy and robust prediction value in predicting 1-year, 3-year, and 5-year OS and DSS outcomes in training, internal validation, and external validation cohorts. Besides, two online tools regarding OS and DSS prediction were established, facilitating nomogram score calculation, risk group determination, as well as survival prediction for each individual patient.

**Conclusion:**

Over the past 40 years, the incidence of CRNENs presented increased steadily, along with improved survival outcomes. Grade III-IV, higher TNM stage, liver metastasis, brain metastasis, and without receiving surgery were found to be associated with worse OS and DSS. Advanced age was a risk factor for OS but not DSS. Nomograms for patients with stage II-IV stage CRNENs are capable of predicting the 1-, 3-, and 5-year OS and DSS rates with high accuracy, and realize risk stratification.

## 1 Introduction

Neuroendocrine neoplasms (NENs) generally refer to all heterogeneous tumors originating from peptidergic neurons and neuroendocrine cells, which can occur in many organs and tissues of the body. The 2019 World Health Organization (WHO) classification of Tumors of the digestive system updated the digestive system NENs classification (1). NENs were divided into neuroendocrine tumors (NET) G1, NET G2, NET G3, neuroendocrine carcinoma (NEC) and mixed neuroendocrine-non-neuroendocrine neoplasm (MiNEN). In recent years, the incidence of gastroenteropancreatic neuroendocrine neoplasms (GEP-NENs) has gradually increased, among which the incidence of colorectal NENs (CRNENs) has increased 10 times (2, 3). It may be due to the popularity of abdominal radiography and endoscopy (3, 4). Most CRNENs are nonfunctional (5), whose clinical symptoms are similar to those of typical colorectal cancer.

CRNENs are more common at stage I, which can often be treated by endoscopy with a good prognosis (6, 7). The European Neuroendocrine Tumor Society (ENETS) and Union for International Cancer Control/American Joint Committee on Cancer (UICC/AJCC) guidelines classify locally advanced and metastatic NENs as stage II-IV. A previous study identified 24.8% of patients with CRNENs at stage II-IV (8). Even though they constitute only a small percentage of diagnosed CRNENs, they pose high mortality risk due to their aggressiveness and high malignancy. Among those patients with a median overall survival (OS) of 9 months to 13.2 months, the 3-year OS rate ranged from 5.9%-36.2%, and the 5-year OS rate ranged from 4.1%- 29.8% (9–11). For CRNENs, the AJCC TNM staging system of 2010 and the WHO’s histological classification system were crucial prognostic systems (12, 13). Nevertheless, there are many other important prognostic factors that may also influence individual outcomes with CRNENs, such as age at diagnosis, sex, race, tumor location, grade, treatment and so on (14–17). Therefore, it is a clinical demand to establish more capable prognostic predictive models for stages II-IV CRNENs. Nomograms are currently widely used for individualized prediction of patient outcomes, and online tools of established nomograms expanded their convenience for clinical application. Different predictors and determinants can be combined to create a nomogram that can generate individuals’ digital probabilities of clinical events, thus meeting our requirements for biological and clinical integrated models and personalized medicine (18).

As there are few CRNENs-specific reports, this study examined the incidence trend for all stage CRNEN patients and identified independent prognostic factors for stage II-IV patients diagnosed from Surveillance, Epidemiology, and End Results (SEER) cancer registry. Following that, we aimed to create prognostic nomograms to provide patients and clinicians with valuable prognostic information about stage II-IV CRNENs and risk factors increasing CRNENs-related death.

## 2 Methods

### 2.1 Datasets and patients

#### 2.1.1 Datasets and respective aims

We assessed the time trend of incidence by extracting the annual incidence of CRNENs from 1975 to 2019 from the SEER Research Plus Data 9 registry. Since our study was limited to the 7th AJCC staging, we only extracted data from the SEER database of stage II-IV CRNENs from January 2010 to December 2016 to investigate survival and prognostic factors. An independent validation cohort of 62 patients who had been diagnosed with CRNENs between January 2010 and January 2016 at the Cancer Hospital, Chinese Academy of Medical Sciences & Peking Union Medical College was also collected for validation. For this study, the patient information was stored in the hospital database with their written consent. This study was exempted from ethical requirements by the ethics committee of Cancer Hospital, Chinese Academy of Medical Sciences.

#### 2.1.2 Inclusion and exclusion criteria

All the required data was extracted using SEER Stat software (version 8.4.0, SEER ID: 12834-Nov2021). Patients with CRNENs should meet the following criteria: i) NENs pathological type according to the International Classification of Diseases for Oncology, 3rd edition (ICD-O3, 8013/3: Large cell neuroendocrine carcinoma, 8041/3: Small cell carcinoma, NOS, 8240/3: Carcinoid tumor, NOS, 8241/3: Enterochromaffin cell carcinoid, 8244/3: Mixed adenoneuroendocrine carcinoma, 8246/3: Neuroendocrine carcinoma, NOS, 8249/3: Atypical carcinoid tumor). ii) Primary tumor of the colorectal site with the corresponding codes C18.0–C18.9. iii) All patients had stage II-IV that was based on the 7th AJCC staging system from 2010 to 2016 (the 7th AJCC staging system was implemented from 2010 to 2016). iv) Complete and accurate data which included age, sex, race, tumor location, pathological type, grade, the 7th AJCC staging, radiotherapy, surgery, chemotherapy, time of death, last follow-up date, and cause of death. Multiple primary or secondary tumors are excluded. Patients with survival time=0 or not available (NA) were excluded.

### 2.2 Study variables and outcomes

Clinical variables including age, sex, incidence, race, location, histology, grade, TNM stage which were according to the 7th editions of the American Joint Committee on Cancer (AJCC) TNM staging system, metastasis information (i.e bone, brain, liver, and lung metastasis), treatment (i.e. surgery, chemotherapy, and radiation). The primary outcome of this study was OS. OS is defined as the time from diagnosis of CRNENs to death of any cause. The secondary outcome of this study was disease-specific survival (DSS) which was defined as the time from diagnosis of CRNENs to the death specifically caused by CRNENs, while the death due to other cause was deemed as a competing risk.

### 2.3 Incidence analysis of CRNENs from 1975 to 2019

The incidence was age-adjusted to the 2000 US Standard Population and was calculated as cases per 1 million people per year from 1975 to 2019. The time trend of CRNENs incidence of different sex and race were also evaluated. The annual percentage changes (APCs) were calculated based on weighted least squares.

### 2.4 Nomogram construction and validation

A total of 668 patients with stage II-IV CRNENs from the SEER database were randomly divided into a training cohort (n=468) and a validation cohort (n=200) at a ratio of 7:3. Next, the training cohort was used for prognostic analysis and competing risk analysis, the validation cohort and the independent validation cohorts were used for internal and external validation.

#### 2.4.1 Cox regression analysis and nomogram establishment for OS

In the prognostic analysis of OS, univariate and multivariate Cox regression analyses were applied, the proportional hazard hypothesis test (PH) was applied to evaluate prognostic factors, and factors with P-values < 0.05 in univariate Cox analysis were added to a multivariate Cox regression model to detect independent prognostic indicators. Based on the results of the multivariate Cox analysis of the training cohort, nomograms were created that incorporated all the independent prognostic factors to predict patients’ risk of living shorter than 1-, 3-, or 5-year. Every variable was linked to a specific point on a horizontal line, the risk score of each patient can be obtained by adding these points together and 1-, 3-, and 5 years OS rates can be anticipated. The individual risk score of each patient in the training cohort was calculated, according to which patients were divided into either low-risk, medium-risk, or high-risk groups by X-tile software (version 3.6.1).

#### 2.4.2 Competing risk analysis and nomogram establishment for DSS

In the competing risk analyses, we used cumulative incidence function (CIF) and Fine-Gray competing risk regression to evaluate the cumulative rate of stage II-IV CRNENs mortality *via* the “cmprsk” R package. The event of interest was defined as death specifically due to CRNENs, whereas the competing risks were defined as death due to other causes or loss to follow up. Factors with P-values < 0.05 in univariate competing risk analysis were added to a multivariate competing risk regression model to detect independent risk indicators of death specifically due to CRNENs.

#### 2.4.3 Nomogram validation of OS and DSS

The predicting outcomes of the model nomogram of OS and DSS were evaluated in the respective training, internal, and external validation cohorts by calibrating curves and the decision-curve analysis (DCA). Besides, we evaluated the accuracy of the nomogram model by examining the 1-, 3-, 5- year receiver operating characteristic (ROC) curve, 5-year variable-dependent ROC curves, and time-dependent area under the ROC curve (AUC). R packages involved in the analysis mentioned above included “survival”, “survminer”, “rms”, “QHScrnomo”, “regplot”, “ggDCA”, and “timeROC”.

#### 2.4.4 Online predictive tools regarding OS and DSS

Two online tools regarding OS and DSS of patients with CRNENs were built to conveniently calculate patient nomogram scores, determine risk group, and predict survival probability using the “DynNom” and “Shiny” R package, as well as the Shiny website (https://www.shinyapps.io/).

### 2.5 Statistical analysis

Baseline categorical variables were described using frequencies with percentages. The optimal cutoff value of continuous variable age was calculated using the “surv_cutpoint” function of the “survminer” R package. Pearson’s chi-square test was used in the comparison of categorical data, while when frequencies were below 5, Fisher exact test was applied. All statistical analyses were performed using R software (version 4.1.0). We used Kaplan-Meier survival estimation along with the log-rank test to estimate the OS and DSS of the whole group of patients. The risk factors of OS and DSS were evaluated by Cox regression analysis and competing risk analysis, respectively. A two-sided P value < 0.05 was considered statistically significant.

## 3 Results

### 3.1 Incidence and patient baseline characteristics

**Figure 1:**
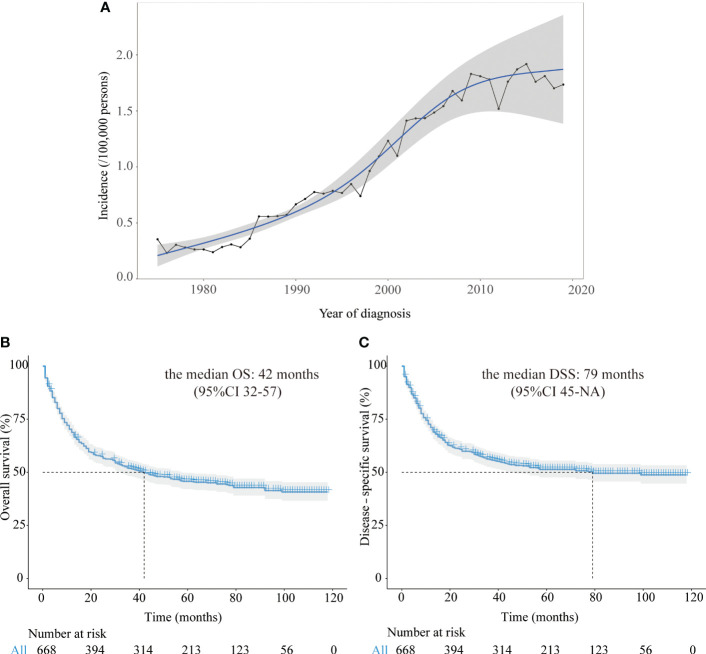
**(A)** The annual age-adjusted incidence of all CRNENs patients from 1975 to 2019. **(B)** Kaplan-Meier curves of OS in CRNENs patients from 2010 to 2016. **(C)** Kaplan-Meier curves of DSS in CRNENs patients from 2010 to 2016. CRNENs, colorectal neuroendocrine neoplasms, OS, overall survival; DSS, disease-specific survival; NA, not available.

From 1975 to 2019, there had been a steady rise in the incidence of CRNENs (**Figure 1A**), which was increased from 0.31 to 1.69 parts per million (annual percentage change [APC]: 4.50; 95% confidence interval [CI]: 3.90–5.11, P < 0.05).

In this study, there were 668 patients with stage II-IV CRNENs recruited from 2010 to 2016 for prognostic analysis, among which males and females accounted for 53.3% and 46.7%, respectively. 20.7% of patients were ≥75 years old. 73.7% of patients were white, 14.5% and 11.8% were black and other races (American Indians/Alaskan Native Americans, Asians or Pacific Islanders and Unknown), respectively. The patients were divided into four groups based on histological grade: well differentiated (G I), moderately differentiated (G II), poorly differentiated (G III) and undifferentiated (G IV). We combined G I and G II into G I/II, G III and G IV into G III/IV for further analyses, which accounted for 48.4% and 51.6%, respectively. 65.1% of the lesion was located in the colon, and 34.9% showed up in the rectum. There were 17.7% of CRNENs patients in stage II, followed by 37.0% in stage III, and 45.4% in stage IV. The majority of patients (72.0%) underwent surgery, followed by 34.9% and 10.0% of them receiving chemotherapy or radiotherapy. The incidence of bone, brain, liver, and lung metastasis was 5.7%, 1.0%, 37.4%, and 5.1%, respectively. Our hospital diagnosed 62 CRNENs patients from 2009 to 2017, whose data was evaluated for independent validation. The baseline demographic features of all patients included in this study were shown in **Table 1**.

**Table 1:** Clinicopathological characteristics of patients with stage II-IV CRNENs.

Variables	Training cohort	Internal validation cohort	P-value	Overall	External validation cohort
	(N=468)	(N=200)		(N=668)	(N=62)
Sex					
Female	222 (47.4%)	90 (45.0%)	0.622	312 (46.7%)	42 (67.7%)
Male	246 (52.6%)	110 (55.0%)		356 (53.3%)	20 (32.3%)
Age					
< 75	376 (80.3%)	154 (77.0%)	0.383	530 (79.3%)	56 (90.3%)
≥75	92 (19.7%)	46 (23.0%)		138 (20.7%)	6 (9.7%)
Race					
Black	75 (16.0%)	22 (11.0%)	0.237	97 (14.5%)	0(0%)
Others*	55 (11.8%)	24 (12.0%)		79 (11.8%)	62(100%)
White	338 (72.2%)	154 (77.0%)		492 (73.7%)	0(0%)
Location					
Colon	302 (64.5%)	133 (66.5%)	0.689	435 (65.1%)	18 (29.0%)
Rectum	166 (35.5%)	67 (33.5%)		233 (34.9%)	44 (71.0%)
Grade					
I/II	244 (52.1%)	101 (50.5%)	0.762	345 (51.6%)	16 (25.8%)
III/IV	224 (47.9%)	99 (49.5%)		323 (48.4%)	46 (74.2%)
Histology					
Atypical carcinoid tumor	11 (2.4%)	9 (4.5%)	0.347	20 (3.0%)	4(6.5%)
Carcinoid tumor	176 (37.6%)	73 (36.5%)		249 (37.3%)	33(53.2%)
Large cell neuroendocrine carcinoma	35 (7.5%)	19 (9.5%)		54 (8.1%)	7(11.3%)
Mixed adenoneuroendocrine carcinoma	30 (6.4%)	7 (3.5%)		37 (5.5%)	6(9.7%)
Neuroendocrine carcinoma	179 (38.2%)	73 (36.5%)		252 (37.7%)	2(3.2%)
Small cell carcinoma	37 (7.9%)	19 (9.5%)		56 (8.4%)	10(16.1%)
Stage					
II	81 (17.3%)	37 (18.5%)	0.725	118 (17.7%)	8 (12.9%)
III	170 (36.3%)	77 (38.5%)		247 (37.0%)	32 (51.6%)
IV	217 (46.4%)	86 (43.0%)		303 (45.4%)	22 (35.5%)
Surgery					
No/Unkonwn	136 (29.1%)	51 (25.5%)	0.398	187 (28.0%)	5 (8.1%)
Yes	332 (70.9%)	149 (74.5%)		481 (72.0%)	57 (91.9%)
Chemotherapy					
No/Unkonwn	303 (64.7%)	132 (66.0%)	0.823	435 (65.1%)	19 (30.6%)
Yes	165 (35.3%)	68 (34.0%)		233 (34.9%)	43 (69.4%)
Radiation					
No/Unkonwn	420 (89.7%)	181 (90.5%)	0.875	601 (90.0%)	51 (82.3%)
Yes	48 (10.3%)	19 (9.5%)		67 (10.0%)	11 (17.7%)
bone metastases					
No	440 (94.0%)	190 (95.0%)	0.749	630 (94.3%)	62(100%)
Yes	28 (6.0%)	10 (5.0%)		38 (5.7%)	0(0%)
brain metastases					
No	463 (98.9%)	198 (99.0%)	1	661 (99.0%)	61 (98.4%)
Yes	5 (1.1%)	2 (1.0%)		7 (1.0%)	1 (1.6%)
liver metastases					
No	287 (61.3%)	131 (65.5%)	0.35	418 (62.6%)	47 (75.8%)
Yes	181 (38.7%)	69 (34.5%)		250 (37.4%)	15 (24.2%)
lung metastases					
No	441 (94.2%)	193 (96.5%)	0.303	634 (94.9%)	62(100%)
Yes	27 (5.8%)	7 (3.5%)		34 (5.1%)	0(0%)
OS month					
median (95CI)	40 (30-57)	72 (39-NA)	/	42 (32-57)	45 (26-NA)
DSS month					
median (95CI)	47 (29-NA)	NA (43-NA)	/	79 (45-NA)	48 (26-NA)

* Others refer to American Indians/Alaskan Native Americans, Asians or Pacific Islanders and Unkonwn.

CRNENs, colorectal neuroendocrine neoplasms; grade: well differentiated (G I), moderately differentiated (G II), poorly differentiated (G III) and undifferentiated (G IV); OS, overall survival; DSS, disease-specific survival; NA, not available.

### 3.2 Survival and prognostic analysis

After a median follow-up time of 74 months (95%CI: 70-78), the median OS was 42 months (95%CI 32-57) among all 668 patients. After a median follow-up time of 68 months (95%CI: 66-73), the median DSS was 79 months (95%CI 45-not available [NA]) among all patients. In our study of the SEER dataset, the reason why NA contains in the 95% CI of DSS and other subgroup survival analyses is that the follow-up time was shorter than the median time of the respective group. The Kaplan-Meier curves of OS and DSS for all patients in the SEER dataset were represented in **Figures 1B, C**.

For further prognostic analysis, the 688 patients with CRNENs were randomly divided into a training cohort (n=468) and a validation cohort (n=200). Patient baseline characteristics between the two cohorts were well-balanced (**Table 1**). The training cohort was used for prognostic analysis and nomogram construction, the validation cohort and an independent validation cohort were used for internal and external validation. The results of univariate and multivariate analysis of OS by Cox regression analysis and DSS by competing risk analysis in the training cohort were summarized in **Table 2**.

**Table 2:** Univariate and multivariate Cox analysis of OS of the II-IV stage patients with CRNENs.

Variables	Univariate analysis		Multivariate analysis	
	HR (95% CI)	P	HR (95% CI)	P
Sex				
Male vs Female	1.24 (0.97-1.58)	0.081	1.25 (0.96-1.63)	0.095
Age				
≥75 vs <75	2.37 (1.81-3.11)	**<0.001**	2.03 (1.5-2.76)	**<0.001**
Race				
Black vs Others*	1.39 (0.85-2.27)	0.196	1.15 (0.69-1.91)	0.601
Black vs White	1.65 (1.13-2.39)	**0.009**	1.11 (0.76-1.64)	0.587
Histology				
Atypical carcinoid tumor vs Carcinoid tumor	2.53 (1-6.4)	0.05	1.12 (0.43-2.91)	0.814
Mixed adenoneuroendocrine carcinoma vs Carcinoid tumor	5.4 (3.22-9.05)	**<0.001**	1.34 (0.68-2.62)	0.397
Neuroendocrine carcinoma vs Carcinoid tumor	4.96 (3.51-7)	**<0.001**	1.23 (0.76-1.99)	0.408
Large cell neuroendocrine carcinoma vs Carcinoid tumor	6.17 (3.8-10.02)	**<0.001**	0.86 (0.44-1.7)	0.669
Small cell carcinoma vs Carcinoid tumor	9 (5.7-14.21)	**<0.001**	1.14 (0.61-2.16)	0.679
Grade				
G III/IV vs G I/II	6.95 (5.23-9.22)	**<0.001**	4.66 (2.92-7.42)	**<0.001**
Location				
Rectum vs Colon	1.17 (0.91-1.5)	0.211	-	-
Stage				
IIII vs II	2.58 (1.47-4.51)	**0.001**	2.22 (1.25-3.94)	0.006
IV vs II	9.01 (5.31-15.31)	**<0.001**	3.99 (2.03-7.83)	**<0.001**
Surgery				
Yes vs No/unkonwn	0.25 (0.2-0.33)	**<0.001**	0.62 (0.44-0.86)	**0.004**
Chemotherapy				
Yes vs No/unkonwn	3.46 (2.69-4.43)	**<0.001**	0.87 (0.63-1.19)	0.379
Radiation				
Yes vs No/unkonwn	1.77 (1.24-2.52)	**0.002**	0.89 (0.59-1.33)	0.559
liver metastases				
Yes vs No	4.05 (3.16-5.18)	**<0.001**	1.61 (1.03-2.51)	**0.037**
brain metastases				
Yes vs No	11.88 (4.77-29.59)	**<0.001**	4.57 (1.66-12.58)	**0.003**
bone metastases				
Yes vs No	2.97 (1.98-4.48)	**<0.001**	0.85 (0.53-1.38)	0.513
lung metastases				
Yes vs No	3.72 (2.46-5.63)	**<0.001**	0.98 (0.59-1.62)	0.94

* Others refer to American Indians/Alaskan Native Americans, Asians or Pacific Islanders and Unkonwn

CRNENs, colorectal neuroendocrine neoplasms; grade: well differentiated (G I), moderately differentiated (G II), poorly differentiated (G III) and undifferentiated (G IV).

#### 3.2.1 Cox regression analysis for OS

In patients with stage II-IV CRNENs, univariate and multivariate Cox regression analyses were used to identify independent prognostic factors of OS. After univariate analysis, variables with a P<0.05 including sex, age, histology, grade, stage, surgery, chemotherapy, radiation, liver metastases, brain metastases, bone metastases, and lung metastases were further investigated in multivariate Cox analysis. After multivariate analysis, age≥75 (HR: 2.03, 95%CI: 1.5-2.76), grade III-IV (HR: 4.66, 95%CI: 2.92-7.42), stage ([stage III vs stage II] HR: 2.22, 95%CI: 1.25-3.94; [stage IV vs stage III] HR: 3.99, 95%CI: 2.03-7.83), liver metastasis (HR: 1.61, 95%CI: 1.03-2.51), brain metastasis (HR: 4.57, 95%CI: 1.66-12.58). In terms of treatment, surgery can significantly prolong the OS of patients with CRNENs (HR: 0.62, 95%CI: 0.44-0.86), but not chemotherapy or radiation (**Table 2**).

#### 3.2.2 Competing risk analysis for DSS

In the univariate competing risk analysis of DSS, factors including age, histology, grade, TNM stage, and treatment (surgery, chemotherapy, and radiation) were found to be associated with the DSS of patients with stage II-IV CRNENs. According to the multivariate analysis results, patients in grade III/IV (HR: 4.79, 95%CI: 4.27-5.31), higher TNM stage ([stage III vs stage II] HR: 2.69, 95%CI: 1.96-3.42; [stage IV vs stage II] HR: 4.96, 95%CI: 4.14-5.78), with liver metastasis (HR: 1.86, 95%CI: 1.39-2.32), and with brain metastasis (HR: 5.01, 95%CI: 4.15-5.87) had higher risk of suffering death specifically due to CRNENs. In terms of treatment, surgery can significantly reduce CRNENs-specific death (HR: 0.67, 95%CI: 0.29-1.05), but not chemotherapy or radiation (**Table 3**).

**Table 3:** Univariate and multivariate competing risk analysis of DSS of the II-IV stage patients with CRNENs.

variable	Univariate analysis		Multivariate analysis	
	HR (95% CI)	P	HR (95% CI)	P
Sex				
Male vs Female	0.74 (0.49-0.99)	0.026	–	–
Age				
≥75 vs <75	1.81 (1.52-2.1)	**<0.001**	1.43 (1.03-1.83)	0.083
Race				
Black vs Others*	0.9 (0.51-1.29)	0.59	–	–
Black vs White	1.43 (1.14-1.72)	0.02	–	–
Histology				
Atypical carcinoid tumor vs Carcinoid tumor	0.53 (0.69-1.75)	0.31	0.86 (0.14-1.87)	0.77
Mixed adenoneuroendocrine carcinoma vs Carcinoid tumor	2 (1.59-2.41)	**<0.001**	1.62 (0.87-2.37)	0.21
Neuroendocrine carcinoma vs Carcinoid tumor	2.19 (1.94-2.44)	**<0.001**	1.22 (0.69-1.75)	0.46
Large cell neuroendocrine carcinoma vs Carcinoid tumor	2.12 (1.69-2.55)	**<0.001**	1.11 (0.32-1.91)	0.79
Small cell carcinoma vs Carcinoid tumor	2.61 (2.28-2.94)	**<0.001**	1.07 (0.37-1.77)	0.85
Grade				
G III/IV vs G I/II	8.52 (8.21-8.83)	**<0.001**	4.79 (4.27-5.31)	**<0.001**
Location				
Rectum vs Colon	1.19 (0.94-1.44)	0.19	–	–
Stage				
III vs II	0.37 (0.06-0.68)	**<0.001**	2.69 (1.96-3.42)	**0.0076**
IV vs II	5.73 b(5.44-6.02)	**<0.001**	4.96 (4.14-5.78)	**<0.001**
Surgery				
No/unknown vs Yes	0.24 (0.01-0.49)	**<0.001**	0.67 (0.29-1.05)	**0.037**
Chemotherapy				
No/unknown vs Yes	4.29 (4.02-4.56)	**<0.001**	1.06 (0.67-1.45)	0.76
Radiation				
No/unknown vs Yes	1.99 (1.66-2.32)	**<0.001**	1.11 (0.72-1.5)	0.59
liver metastases				
Yes vs No	5 (4.73-5.27)	**<0.001**	1.86 (1.39-2.32)	**0.0089**
brain metastases				
Yes vs No	11.18 (10.69-11.67)	**<0.001**	5.01 (4.15-5.87)	**<0.001**
bone metastases				
Yes vs No	3.1 (2.75-3.45)	**<0.001**	0.87 (0.4-1.34)	0.55
lung metastases				
Yes vs No	3.74 (3.39-4.09)	**<0.001**	0.88 (0.4-1.36)	0.59

* American Indians/Alaskan Native Americans, Asians or Pacific Islanders and Unkonwn.

CRNENs, colorectal neuroendocrine neoplasms; grade: well differentiated (G I), moderately differentiated (G II), poorly differentiated (G III) and undifferentiated (G IV).

### 3.3 Developing nomograms and risk stratification

**Figure 2:**
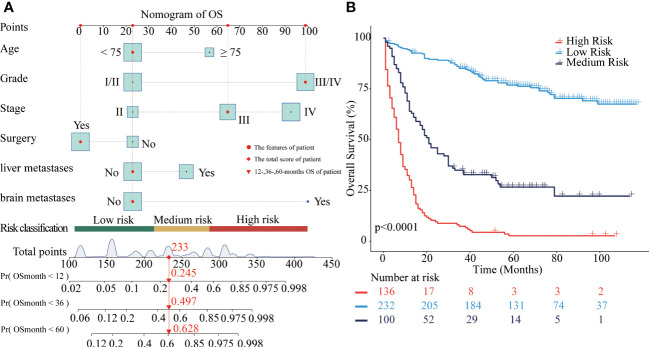
Nomograms to predict OS for patients with CRNENs and risk stratification. **(A)** nomogram of OS. **(B)** the Kaplan-Meier curve of the high-risk, medium-risk, and low-risk groups. CRNENs, colorectal neuroendocrine neoplasms; OS, overall survival.

In the training cohort, independent prognostic factors were used to construct the nomogram of OS for predicting patients’ risk of living shorter than 1-, 3-, or 5-year (**Figure 2A**). After the risk score of each of the 688 patients from the SEER dataset was obtained according to the nomogram, X-tile software was used to divide the patients into low-, medium-, and high-risk groups for OS prediction. The cut-off points of the three risk groups are 214 and 284. As presented in **Figure 2B**, the survival outcomes of OS were significantly different in the three risk groups (P<0.0001). The median OS of the high-risk, medium-risk, and low-risk groups were 7 months (95%CI 6-9), 21 months (95%CI 17-30), and not reached (95%CI NA-NA), respectively. The 5-year OS rates of the three risk groups were 2.6% (95%CI 0.9-7.6), 26.7% (95%CI 18.8-37.7), and 76.9% (95%CI 71.5-82.7), respectively.

**Figure 3:**
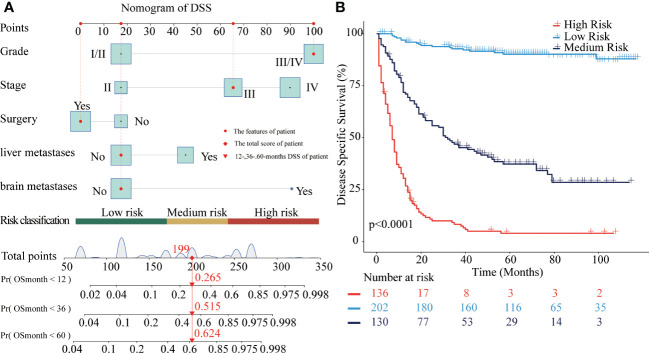
Nomograms to predict DSS for patients with CRNENs and risk stratification. **(A)** nomogram of DSS. **(B)** the Kaplan-Meier curve of the high-risk, medium-risk, and low-risk groups. CRNENs, colorectal neuroendocrine neoplasms; DSS, disease-specific survival.

**Figure 4:**
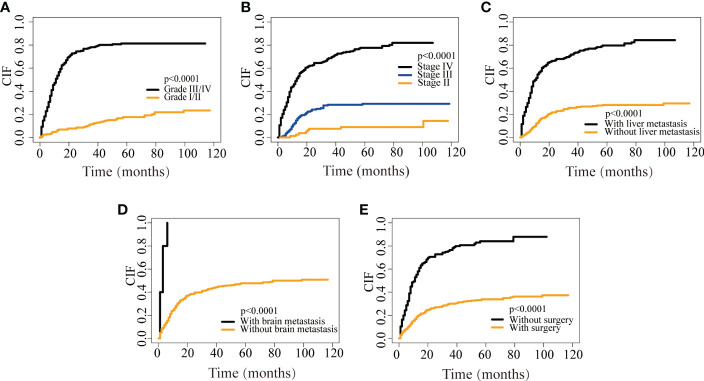
CIF curves depicting CRNENs-caused death based on grade **(A)**, stage **(B)**, liver metastasis **(C)**, brain metastasis **(D)**, and surgery **(E)**. CRNENs, colorectal neuroendocrine neoplasms; CIF, cumulative incidence function.

Similarly, the nomogram of DSS for predicting CRNENs’ specific death of less than 1-, 3-, and 5-year. As presented in **Figure 3A**, the cut-off points of the low-, medium-, and high-risk groups are 169 and 241. As presented in **Figure 3B**, the risk of CRNENs’ specific death increased significantly as the risk increased (P<0.0001). The median DSS of the high-risk, medium-risk, and low-risk groups were 8 months (95%CI 7-9), 47 months (95%CI 33-79), and not reached (95%CI NA-NA), respectively. The 5-year DSS rates of the three risk groups were 5.5% (95%CI 2.7-11.1), 44.8% (95%CI 36.9-54.4), and 96.0% (95%CI 92.9-99.2), respectively. The cumulative hazard function curves of the significant variables with the DSS of patients with stage II-IV CRNENs were visualized in **Figure 4**.

The median time, 1-, 3-, and 5-year rates of the three risk groups for OS and DSS were summarized in **Table 4**.

**Table 4:** Risk stratification for the nomogram of OS and DSS.

Risk group		median time	1-year rate	3-year rate	5-year rate
**OS**	**high risk**	7 months (95%CI 6-9)	30.1% (95%CI 23.3 - 38.9)	7.4% (95%CI 4.0 - 13.4)	2.6% (95%CI 0.9-7.6)
	**medium risk**	21 months (95%CI 17-30)	64.0% (95%CI 55.3 - 74.1)	35.0% (95%CI 26.8 - 45.7)	26.7% (95%CI 18.8-37.7)
	**low risk**	not reached (95%CI NA-NA)	93.9% (95%CI 90.9 - 97.1)	85.2% (95%CI 80.7 - 89.9)	76.9% (95%CI 71.5-82.7)
**DSS**	**high risk**	8 months (95%CI 7-9)	34.6% (95%CI 27.9 - 43.1)	97.5% (95%CI 95.2-100)	5.5% (95%CI 2.7-11.1)
	**medium risk**	47 months (95%CI 33-79)	77.1% (95%CI 70.5 - 84.3)	55.0% (95%CI 47.4-63.8)	44.8% (95%CI 36.9-54.4)
	**low risk**	not reached (95%CI NA-NA)	98.0% (95%CI 95.7-100)	97.5% (95%CI 95.2-100)	96.0% (95%CI 92.9-99.2)

OS, overall survival; DSS, disease-specific survival; NA, not available.

### 3.4 Validation of the nomograms of OS and DSS

**Figure 5:**
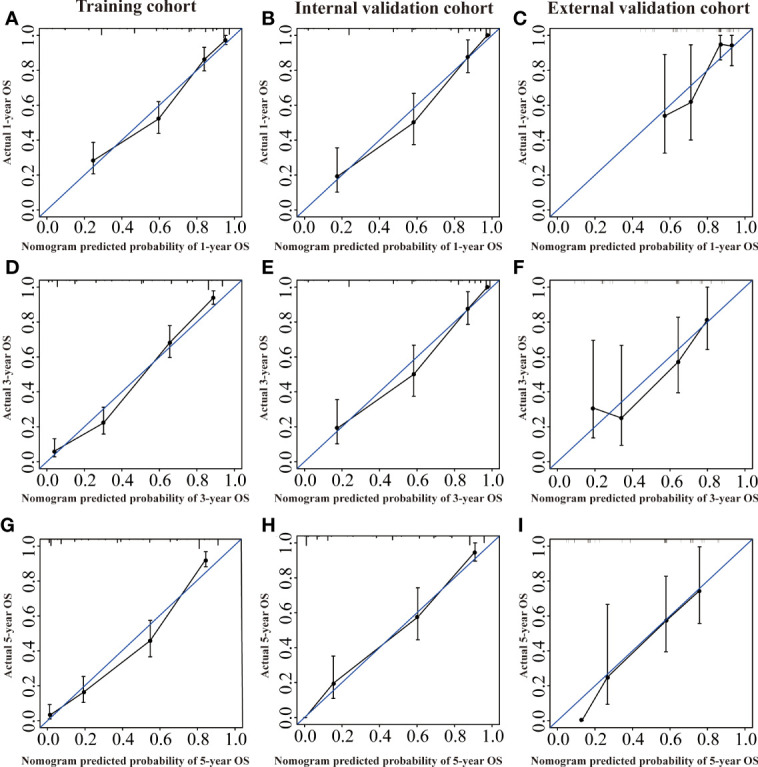
Calibration curves of the nomogram of CRNENs for 1, 3, and 5-year OS rates in the training cohort **(A, D, G)**, the internal validation cohort **(B, E, H)**, and the external validation cohort **(C, F, I)**. CRNENs, colorectal neuroendocrine neoplasms; OS, overall survival.

**Figure 6:**
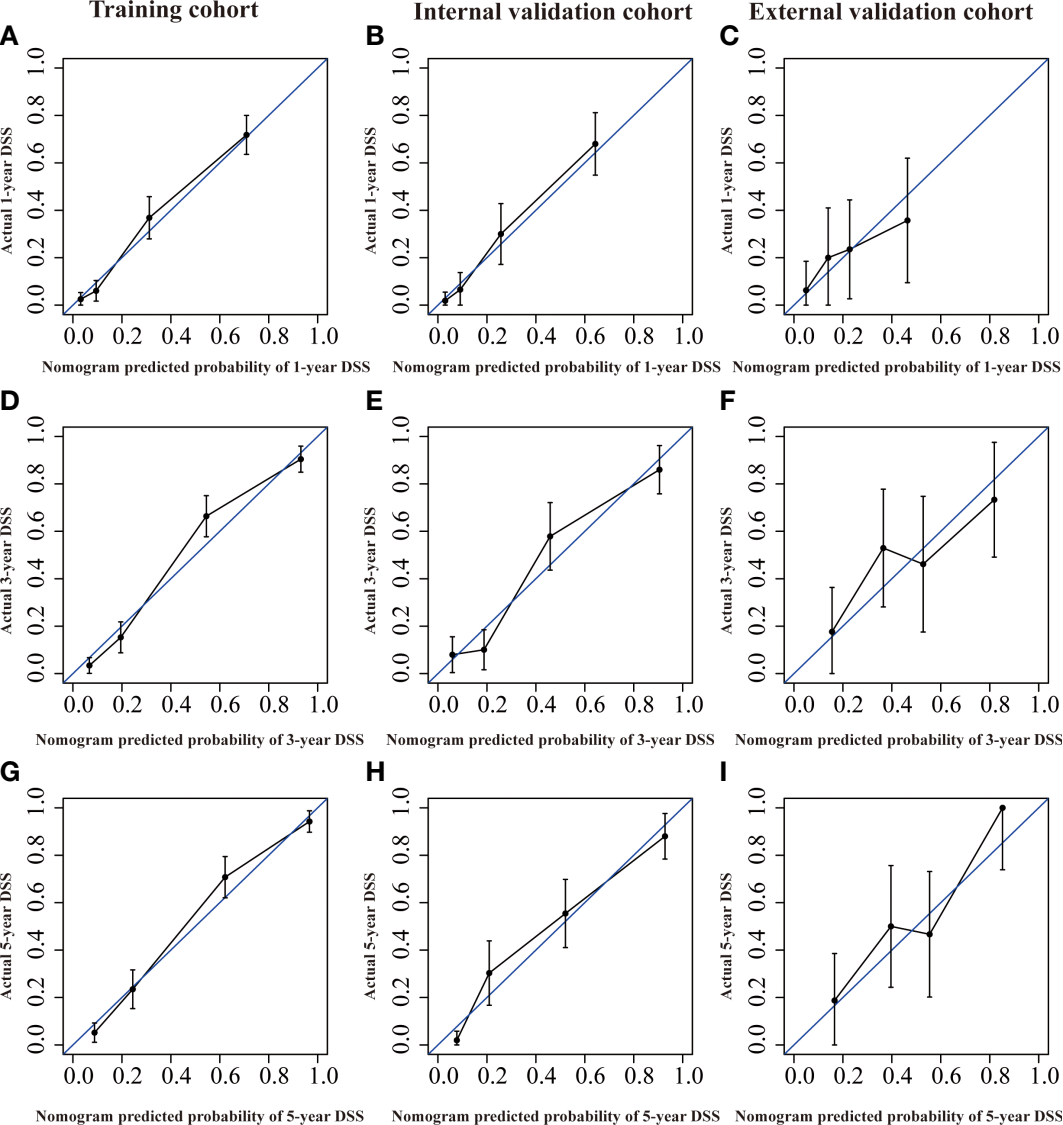
Calibration curves of the nomogram of CRNENs for 1, 3, and 5-year DSS rates in the training cohort **(A, D, G)**, the internal validation cohort **(B, E, H)**, and the external validation cohort **(C, F, I)**. CRNENs, colorectal neuroendocrine neoplasms; DSS, disease-specific survival.

**Figure 7:**
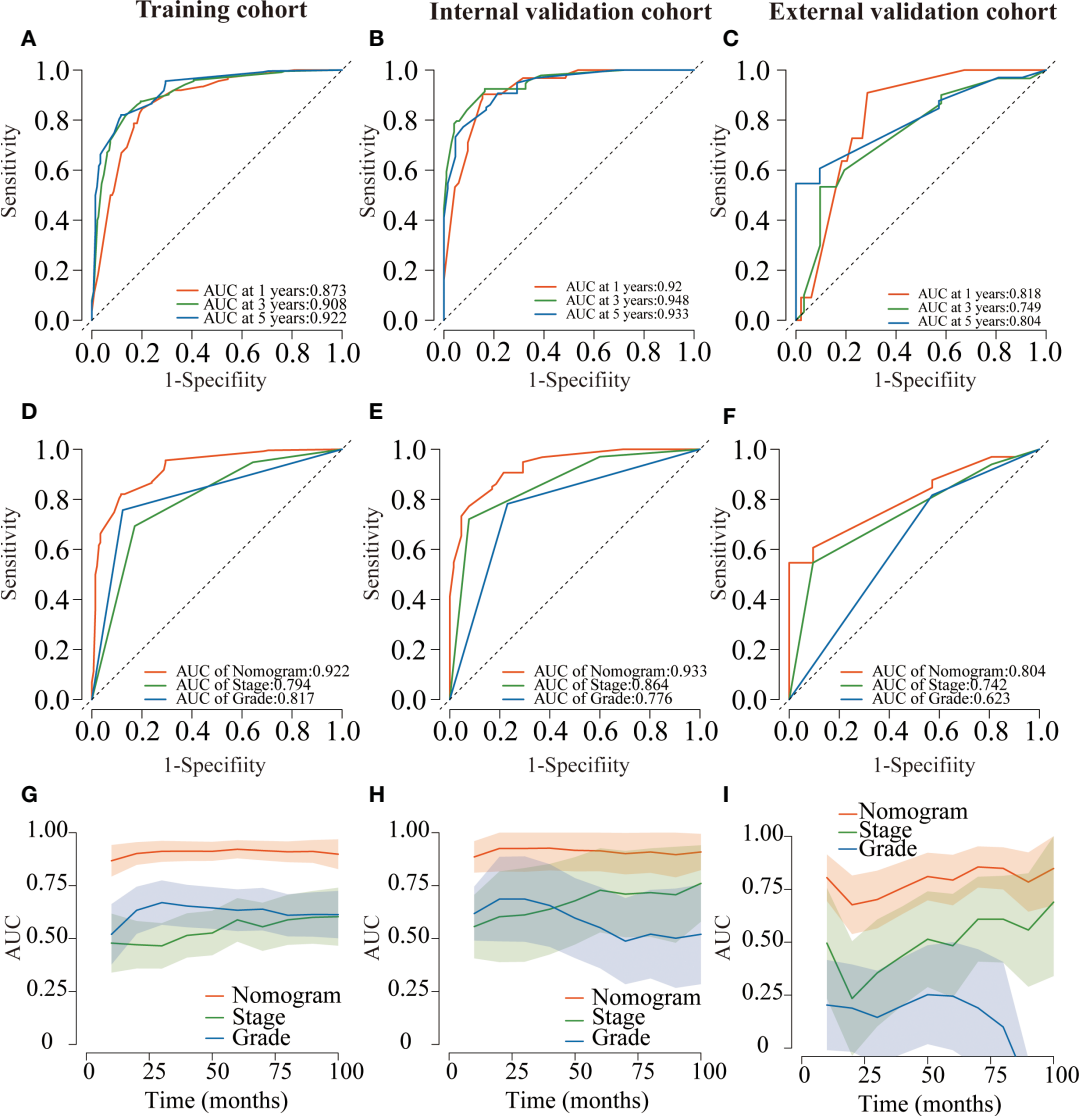
ROC curves for the nomogram of CRNENs for 1, 3, and 5-year OS rates in the training cohort **(A)**, the internal validation cohort **(B)**, and the external validation cohort **(C)**. Variable-dependent ROC curves of CRNENs of 5-year OS in the training cohort **(D)**, the internal validation cohort **(E)**, and the external validation cohort **(F)**. Time-dependent AUC curves for the nomogram of OS in the training cohort **(G)**, the internal validation cohort **(H)**, and the external validation cohort **(I)**. CRNENs, colorectal neuroendocrine neoplasms; OS, overall survival. ROC, receiver operating characteristic; AUC, area under the curve.

**Figure 8:**
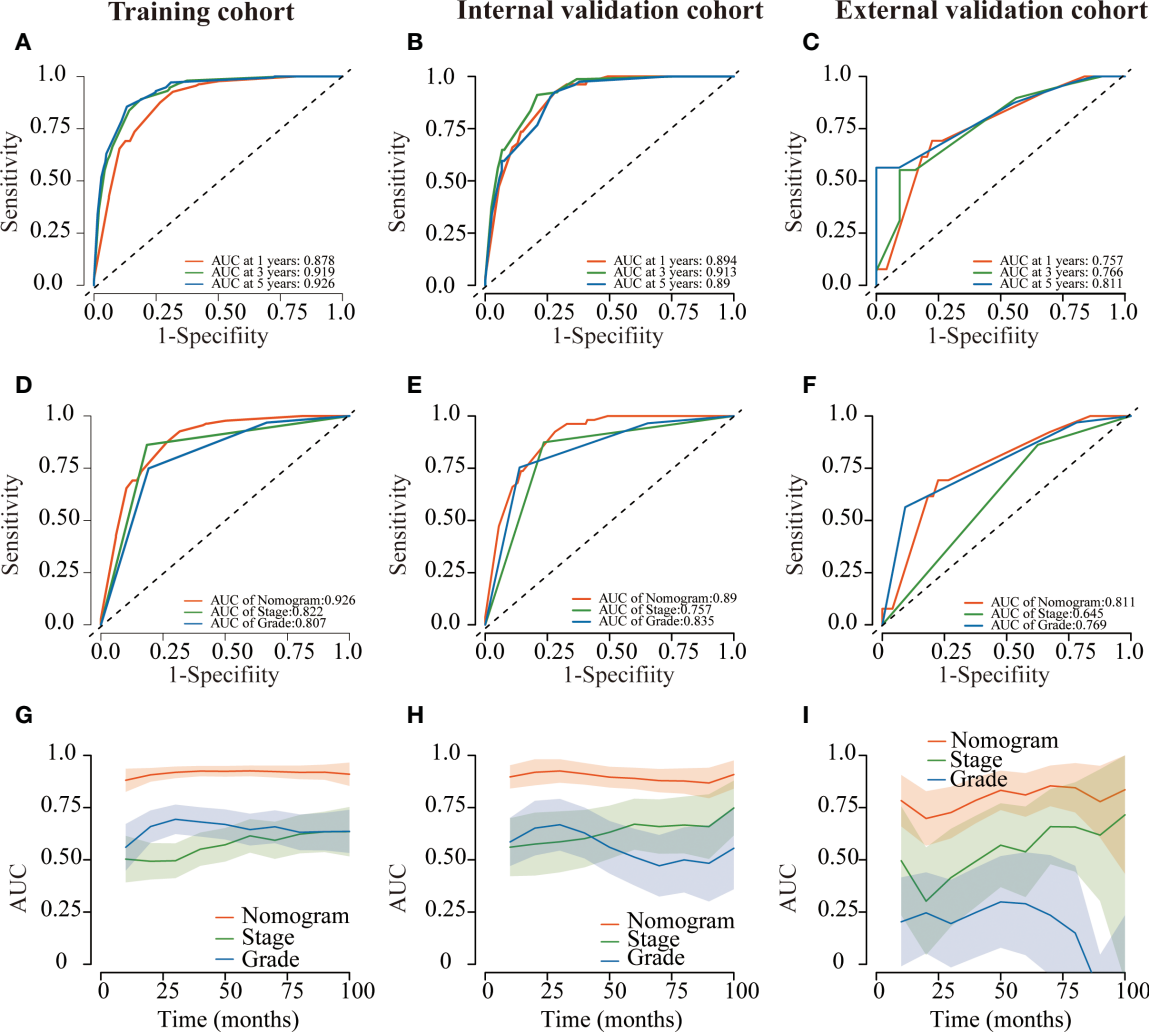
ROC curves of the nomogram of CRNENs for 1, 3, and 5-year DSS rates in the training cohort **(A)**, the internal validation cohort **(B)**, and the external validation cohort **(C)**. Variable-dependent ROC curves of CRNENs of 5-year DSS in the training cohort **(D)**, the internal validation cohort **(E)**, and the external validation cohort **(F)**. Time-dependent AUC curves for the nomogram of DSS in the training cohort **(G)**, the internal validation cohort **(H)**, and the external validation cohort **(I)**. CRNENs, colorectal neuroendocrine neoplasms; DSS, disease-specific survival; ROC, receiver operating characteristic; AUC, area under the curve.

Several validation methods have demonstrated the stability and efficacy of the established nomograms. The calibration plots in the training, internal validation, and external validation cohorts for 1-, 3-, and 5-year OS and DSS were described in **Figures 5**, **6**. The 1-, 3-, and 5-year ROC curves for OS in the three cohorts were described in **Figures 7A–C**. Besides, the variable-dependent 5-year ROC which included nomogram, grade, and stage showed a superior predictive value of the nomogram than the commonly used risk factors grade and stage (**Figures 7D–F**). Time-dependent AUC curves of OS were presented in **Figures 7G–I**. The 1-, 3-, and 5- year ROC curves, variable-dependent ROC of 5-year DSS and time-dependent AUC curves of the DSS nomogram were displayed in **Figures 8A–I**.

**Figure 9:**
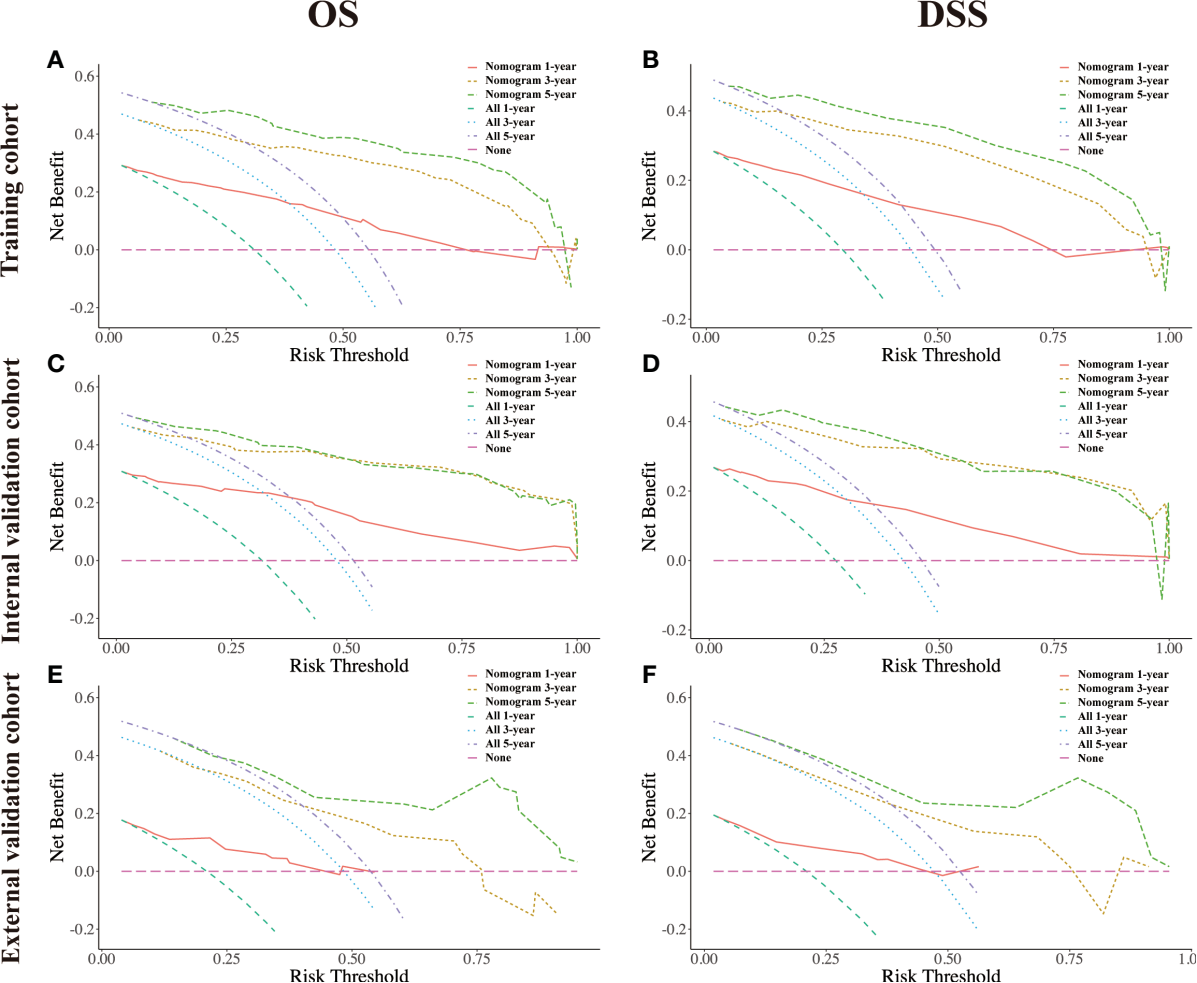
The decision-curve analysis (DCA) plots of the nomogram of CRNENs for 1, 3, and 5-year OS rates in the training cohort **(A)**, the internal validation cohort **(C)**, and the external validation cohort **(E)**. The decision-curve analysis (DCA) plots of the nomogram of CRNENs for 1, 3, and 5-year DSS rates in the training cohort **(B)**, the internal validation cohort **(D)**, and the external validation cohort **(F)**. CRNENs, colorectal neuroendocrine neoplasms; OS, overall survival; DSS, disease-specific survival. Note: “All” refers to intervention for all, and “None” refers to intervention for none. Intervention is considered to be any behavioral or external factor considered by high-risk patients when obtaining positive results from the model.

The DCA plots at 1-, 3-, and 5- year rates of OS and DSS in the training, internal validation, and external validation cohorts illustrated that our nomogram for CRNENs achieved positive net clinical benefits at a broad range of threshold probabilities, which indicated a high clinical utility for the nomogram model (**Figure 9**). Furthermore, two online tools specifically for OS (https://crnens-os-prediction.shinyapps.io/dynnomapp/) and DSS (https://crnens-dss-prediction.shinyapps.io/dynnomapp/) were built to conveniently realize nomogram score calculation, risk group determination, as well as survival prediction for each individual patient.

## 4 Discussion

The CRNENs are heterogeneous neoplasms characterized by neuroendocrine secretory granules in the cytoplasm of the cells, but its pathogenesis is still unclear. As little information is available on the incidence and survival of CRNENs, it is difficult to assess its prognosis. In this study, we assessed the incidence and survival outcomes of patients with stage II-IV CRNENs from both the SEER database and external real-world cohort as well as developed relevant prognostic nomograms and convenient online tools for OS and DSS prediction.

In our study of the SEER dataset, even though it is a rare disease, the incidence of CRNENs increased from 0.31 to 1.69 parts per million over the past 44 years. 20.7% of patients with CRNENs were 75 years old or older. We observed that the prognosis of OS in elderly patients was poor, while the impact of age on CRNENs’ specific death failed to show significance. In the SEER database, the grade is classified as highly differentiated (G I), moderately differentiated (G II), poorly differentiated (G III), and undifferentiated (G IV), which are determined primarily according to the level of ki-67 index and are deemed as important prognostic factors of OS and DSS (19, 20). NET G3 and NEC were highly invasive and had a poor prognosis. More than half of the patients had distant metastasis at the time of diagnosis (9, 21). In this study, we also explored the prognostic value of specific metastatic location for CRNENs, which showed that brain and liver metastasis were independent risk indicators of shorter OS outcome and would increase the risk of CRNENs-specific death, while bone and lung metastasis failed to show significance in multivariate analysis. Brain metastasis in neuroendocrine tumors is rare with an incidence of 1.5-5% (22). In our analysis, brain metastasis was only observed in 1.0% of patients with stage II-IV CRNENs. While liver metastasis was the most common metastatic organ which was consistent with our study. In another study, liver metastasis was observed in 72.84% of stage IV gastrointestinal neuroendocrine neoplasm and was associated with a higher risk of concurrent, while the significance of brain metastasis failed to be observed (23). We also found that 65.7% of the stage II-IV CRNENs occurred in the colon, but the location of the tumor had no correlation with the prognosis, which was different from the results previously reported (24, 25). Many patients were initially diagnosed with CRNENs in an advanced stage, our study showed that 45.4% of patients were in stage IV, which was similar to the previous study (26). Our study also confirmed the robust prognostic ability of the TNM staging system in CRNENs.

Recently, competing risk analyses have received increasing higher popularity in the clinical research of cancer-specific death (27). Compared to traditional Cox regression analysis, it introduced the concept of competitive risk and avoid the impact of death due to other causes to the final result (28). In this study, the competing risk model was used to analyze the prognostic factors of death specifically due to CRNENs. Our results showed that, except age, all other risk factors of OS including grade, stage, surgery, brain metastasis, and liver metastasis were found to increase the risk of CRNENs-specific death. The nomogram of DSS and relative online tools were also established successfully.

In terms of treatment, surgery can improve the prognosis of stage II-IV stage CRNENs. Although radiotherapy and chemotherapy have always been the treatment for patients with unresectable primary tumors (29), our analysis showed that they did not significantly prolong the OS outcomes of patients with CRNENs or decrease the risk of CRNENs-specific death. The treatment of CRNENs is determined according to the degree of differentiation and staging of the tumor. CRNENs with a diameter of less than 1 cm, being limited to the submucosa and without distant metastasis, can be resected locally under an endoscope or through the anus (30, 31). As a result of the risk of regional lymph node metastasis, radical surgery is recommended for CRNENs with locally advanced stages (32). However, the therapeutic value of surgery is still debated in patients with advanced tumors. In previous studies, palliative resection has been suggested to be a viable option for patients experiencing obstructions, bleeding, perforations, etc (31). Nevertheless, many studies suggested that palliative surgery can improve survival rates of CRNENs by reducing tumor burden (11, 21, 33). Moreover, simultaneous surgical treatment is recommended for patients with liver metastasis in order to improve long-term survival (29, 34). These are consistent with our findings. The following are some possible reasons. To begin with, the efficacy of chemotherapy for advanced G1, G2, G3 and NEC is unsatisfying with an objective response rate of less than 50%, and the options for chemotherapeutic agents were rather limited (29, 35). Secondly, the use of radiotherapy is only recommended for patients with locally advanced and advanced rectal neuroendocrine tumors (36, 37). It is believed that conventional radiotherapy can reduce the local recurrence rate but will not improve survival rates (37). Thirdly, confounding factors existed inevitably in this study, due to the inclusion of all pathological classifications, grades, and stages of CRNENs, the sensitivity of radiotherapy and chemotherapy might vary according to the stage, grade, and location of the tumor. In this study, the detailed radiation modality is unavailable for us to perform further analysis. In previous studies, the effectiveness of peptide receptor radionuclide therapy (PPRT) has been reported to have an impact on the prognosis of CRNENs (38, 39).

In recent years, some novel treatments such as somatostatin analogue (SSA) and targeted therapy are found to show efficacy in the treatment of CRNENs. SSA has been proven to inhibit tumor growth and prolong tumor progression-free survival (40). The effect of SSA in the treatment of NENs is mainly to control symptoms and stabilize tumor progression. SSA acts on somatostatin receptors on the surface of tumor cells to inhibit functional NENs secreting peptides and growth hormone (41, 42). Although the mammalian target of rapamycin (mTOR) inhibitor everolimus and multi-targeted kinase inhibitor sunitinib has been approved for locally advanced and metastatic pancreatic NENs in clinical practice (43), the effect of its application in colorectal neuroendocrine tumors still needs to be observed (44).

Some limitations remained in this study. Firstly, the SEER database lacks neuroendocrine biomarkers information like chromogranin A (CgA), synaptophysin (Syn), and CD56, which are important prognostic factors (45). In addition, apart from the basic treatment records, the SEER database does not contain any additional information about operation mode, chemotherapy regimen, radiation dose, tumor shape, various health statuses, or socioeconomic factors that might influence survival (35, 46). Since these parameters could not be evaluated and integrated into our nomogram, further studies should try to include these valuable factors and assess their values. Secondly, due to the retrospective nature of this study, there is the possibility of selection bias among the patients we enrolled. Larger prospective studies are warranted to verify our results.

## 5 Conclusion

Over the past 40 years, the incidence of CRNENs increased steadily, along with improved survival outcomes. Grade III-IV, higher TNM stage, liver metastasis, brain metastasis, and without receiving surgery were found to be associated with worse OS and DSS. Advanced age was a risk factor for OS but not DSS. Nomograms for patients with stage II-IV stage CRNENs are capable of predicting the 1-, 3-, and 5-year OS and DSS with high accuracy, and realize risk stratification. Besides, two online tools regarding OS and DSS prediction were established, facilitating nomogram score calculation, risk group determination, as well as survival prediction for each individual patient.

## Data availability statement

The original contributions presented in the study are included in the article/supplementary material. Further inquiries can be directed to the corresponding author.

## Ethics statement

The studies involving human participants were reviewed and approved by the ethics committee of the Cancer Hospital of the Chinese Academy of Medical Sciences & Peking Union Medical College. The patients/participants provided their written informed consent to participate in this study. Written informed consent was obtained from the individual(s) for the publication of any potentially identifiable images or data included in this article.

## Author contributions

QL contributed to the conception and design of the study. FZ, LH, and ZW analyzed the data and drafted the manuscript. FW and TX checked all data and results. All authors contributed to the article and approved the submitted version.
